# Supplementary material for: Impact of Belgian COVID-19 lockdown restrictions on autistic individuals’ socio-communicative behaviors and their parents’ quality of life
Source: PLoS One. 2022 Aug 31;17(8):e0273932. doi: 10.1371/journal.pone.0273932 (PMC9433112; doi:10.1371/journal.pone.0273932)
Supplement: S1 File — (DOCX) [file pone.0273932.s001.docx]

**Supplementary material**

**S1 Table. Summary of linear regression models on the nonfunctional socio-communicative behaviors index. Statistically significant simple effects discussed in the manuscript appear in bold fond.**

| Domain 1: Home characteristics | | | | | Domain 2: Autistic individuals characteristics | | | | |
| --- | --- | --- | --- | --- | --- | --- | --- | --- | --- |
| (F(7, 201) = 1.7, *p* = .11, R2 = .06) | | | | | (F(6, 202) = 2.74, *p* = .01, R2 = .07) | | | | |
|  | ꞵ | SE | t value | 95% CI |  | ꞵ | SE | t value | 95% CI |
| Intercept | 3.63 | .24 | 14.980 | 3.15, 4.11 | Intercept | 3.34 | .4 | 8.25 | 2.54, 4.14 |
| Number of cohabitants | .004 | .07 | .06 | -.13, .14 | Gender Male | .09 | .12 | .8 | -.14, .33 |
| Number of minor cohabitants | .03 | .07 | .5 | -.1, .17 | Age 3 to 7 | .14 | .39 | .35 | -.63, .9 |
| Bedrooms missing Yes | .1 | .13 | .8 | -.15, .36 | Age 7 to 18 | -.22 | .39 | -.56 | -.98, .54 |
| Type of housing Semi-detached house | -.2 | .18 | -1.14 | -.56, .15 | Age More than 18 | -.07 | .41 | -.18 | -.88, .73 |
| Type of housing Detached House | -.51 | .21 | -2.4 | -.93, -.09 | **Amount of comorbidities** | **.11** | **.04** | **2.69** | **.03, .18** |
| Outside space at home Big | .06 | .24 | .25 | -.41, .54 | Education Special education school | .08 | .11 | .66 | -.15, .3 |
| Outside space at home Small | -.16 | .23 | -.69 | -.6, .29 |  |  |  |  |  |
| Domain 3: Resources | | | | | Domain 4: Home strategies | | | | |
| (F(5, 203) = 4.68, *p* < .001, R2 = .1) | | | | | (F(6, 202) = 2.57, *p* = .02, R2 = .07) | | | | |
|  | ꞵ | SE | t value | 95% CI |  | ꞵ | SE | t value | 95% CI |
| Intercept | 3.47 | .33 | 10.49 | 2.82, 4.12 | Intercept | 3.65 | .23 | 15.94 | 3.2, 4.11 |
| **Parents’ access to respite care** | **-.19** | **.05** | **-4** | **-.3, -.1** | Homework from school Yes | -.08 | .11 | -.7 | -.29, .14 |
| Financial worries during lockdown | .12 | .07 | 1.85 | .01, .25 | Implementation of homeschooling Yes | .23 | .12 | 1.9 | -.01, .48 |
| Partner’s support | -.02 | .06 | -.3 | -.13, .09 | Schedule at home New schedule | -.21 | .14 | -1.48 | -.49, .07 |
| Professional support | -.01 | .05 | -.21 | -.1, .08 | Schedule at home No schedule | -.23 | .13 | -1.77 | -.49, .03 |
| Parents’ group support | .04 | .06 | .64 | -.08, .16 | **Implementation of rules at home** | **-.12** | **.05** | **-2.13** | **-.23, -.01** |
|  |  |  |  |  | Implementation of ASD strategies | .1 | .06 | 1.57 | -.02, .22 |

**S2 Table. Summary of linear regression models on the quality of life index. Statistically significant simple effects discussed in the manuscript appear in bold fond.**

| Domain 1: Home characteristics | | | | | Domain 2: Autistic individuals characteristics | | | | |
| --- | --- | --- | --- | --- | --- | --- | --- | --- | --- |
| (F(9, 199) = 1.05, *p* = .4, R2 = .04) | | | | | (F(6, 202) = 1. 35, *p* = .24, R2 = .04) | | | | |
|  | ꞵ | SE | t value | 95% CI |  | ꞵ | SE | t value | 95% CI |
| Intercept | 4.31 | .36 | 11.94 | 3.6, 5.03 | Intercept | 3.89 | .51 | 7.68 | 2.89, 4.89 |
| Number of cohabitants | -.11 | .09 | -1.19 | -.29, .07 | Gender Male | .06 | .15 | .39 | -.23, .35 |
| Number of minor cohabitants | -.02 | .09 | -.18 | -.19, .16 | Age 3 to 7 | -.51 | .49 | -1.04 | -1.47, .45 |
| Bedrooms missing Yes | .31 | .17 | 1.84 | -.02, .64 | Age 7 to 18 | -.6 | .48 | -1.25 | -1.56, .35 |
| Type of housing Semi-detached house | -.08 | .22 | -.34 | -.52, .36 | Age More than 18 | -.45 | .51 | -.9 | -1.46, .56 |
| Type of housing Detached House | -.11 | .26 | -.42 | -.63, .41 | Amount of comorbidities | .1 | .05 | 2.01 | .002, .2 |
| Outside space at home Big | -.28 | .3 | -.94 | -.88, .31 | Education Special education school | .18 | .14 | 1.22 | -.11, .46 |
| Outside space at home Small | -.33 | .28 | -1.18 | -.89, .22 |  |  |  |  |  |
| Family composition Single-parent family | -.06 | .21 | -.29 | -.47, .35 |  |  |  |  |  |
| Family composition Stepfamily | -.29 | .25 | -1.16 | -.77, .2 |  |  |  |  |  |
| Domain 3: Resources | | | | | Domain 4: Home strategies | | | | |
| (F(6, 202) = 7.63, *p* < .001, R2 = .18). | | | | | (F(6, 202) = .59, *p* = .74, R2 = .02) | | | | |
|  | ꞵ | SE | t value | 95% CI |  | ꞵ | SE | t value | 95% CI |
| Intercept | 3.56 | .4 | 8.94 | 2.78, 4.35 | Intercept | 3.7 | .29 | 12.8 | 3.14, 4.28 |
| **Parents’ access to respite care** | **-.31** | **.06** | **-5.61** | **-.42, -.2** | Homework from school Yes | -.14 | .14 | -.1.04 | -.42, .13 |
| Financial worries during lockdown | .13 | .08 | 1.65 | -.02, .28 | Implementation of homeschooling Yes | -.05 | .15 | -.31 | -.35, .26 |
| Partner’s support | -.02 | .07 | -.34 | -.15, .11 | Schedule at home New schedule | -.13 | .18 | -.76 | -.49, .22 |
| Professional support | -.03 | .06 | -.54 | -.14, .08 | Schedule at home No schedule | -.05 | .16 | -.3 | -.37, .27 |
| Parents’ group support | .14 | .07 | 1.9 | -.005, .28 | Implementation of rules at home | -.05 | .07 | -.61 | -.18, .09 |
| Contacts with friends during lockdown | .01 | .05 | .2 | -.09, .11 | Implementation of ASD strategies | .08 | .08 | 1.01 | -.07, .23 |
