# Supplementary material for: Impact of Belgian COVID-19 lockdown restrictions on autistic individuals’ socio-communicative behaviors and their parents’ quality of life
Source: PLoS One. 2022 Aug 31;17(8):e0273932. doi: 10.1371/journal.pone.0273932 (PMC9433112; doi:10.1371/journal.pone.0273932)
Supplement: Attachment — Submitted filename: Response to Reviewers.docx [file pone.0273932.s002.docx]

We would like to begin by thanking the editor for giving us the opportunity to revise our manuscript and the reviewers for their comments and corrections. In this document, you will find our responses to the editor’s and the reviewers’ comments. We have addressed all queries, starting with Editor on page 2, Reviewer 1’s on page 6 and Reviewer 2’s on page 10. We repeat each query, one by one, before explaining how it is reflected in the revised version of our manuscript.

1. Journal requirements

*Comment 1*

Please ensure that your manuscript meets PLOS ONE's style requirements, including those for file naming. The PLOS ONE style templates can be found at

https://journals.plos.org/plosone/s/file?id=wjVg/PLOSOne_formatting_sample_main_body.pdf and

https://journals.plos.org/plosone/s/file?id=ba62/PLOSOne_formatting_sample_title_authors_affiliations.pdf.

The revised manuscript now thoroughly meets PLOS ONE’s style requirements.

*Comment 2*

Thank you for stating the following financial disclosure:

“This work is supported by various sources of funding. MW and PD are supported by research grants from the Support Fund Marguerite-Marie Delacroix. PM is supported by a doctoral grant from the ROGER DE SPOELBERCH Foundation. MK is a 2019-2021 Francqui Foundation Research Professor”

Please state what role the funders took in the study. If the funders had no role, please state: ""The funders had no role in study design, data collection and analysis, decision to publish, or preparation of the manuscript.""

If this statement is not correct you must amend it as needed.

Please include this amended Role of Funder statement in your cover letter; we will change the online submission form on your behalf.

Thank you. The funders did not have any role in study design, data collection and analysis, decision to publish, or preparation of the manuscript. This information is included in the cover letter. In addition, we noticed a small error in the Funding statement. MK is actually a 2019-2022 Francqui Foundation Research Professor. This was corrected in the new Funding statement provided in the cover letter.

*Comment 3*

Thank you for stating the following in the Acknowledgments Section of your manuscript:

“We express our gratitude to all the families that took part in this study sharing their experience during this hard time. We also thank Eric Willaye, the SUSA Foundation director, for insights and support throughout the research, as well as Aurore Delhommeau, Aurélie Hanquet and Lucie Ducloux for the help in designing the questionnaire.”

We note that you have provided funding information that is not currently declared in your Funding Statement. However, funding information should not appear in the Acknowledgments section or other areas of your manuscript. We will only publish funding information present in the Funding Statement section of the online submission form.

Please remove any funding-related text from the manuscript and let us know how you would like to update your Funding Statement. Currently, your Funding Statement reads as follows:

“This work is supported by various sources of funding. MW and PD are supported by research grants from the Support Fund Marguerite-Marie Delacroix. PM is supported by a doctoral grant from the ROGER DE SPOELBERCH Foundation. MK is a 2019-2021 Francqui Foundation Research Professor”

Please include your amended statements within your cover letter; we will change the online submission form on your behalf.

The SUSA Foundation is a referral center for the diagnosis of autism in Belgium, not a foundation that financially supports scientific research. They have supported our study by giving us access to their database to disseminate the survey among potential participants. They have not funded the study in any way. Their contribution to our study is small, that’s why they appear in the Acknowledgments section.

However, we did make one small modification to our Acknowledgments section. We wanted to acknowledge the contribution of a colleague who proofread the last version of our manuscript. As a result, our modified Acknowledgments are included in the cover letter and at the end of the revised manuscript.

*Comment 4*

In your Data Availability statement, you have not specified where the minimal data set underlying the results described in your manuscript can be found. PLOS defines a study's minimal data set as the underlying data used to reach the conclusions drawn in the manuscript and any additional data required to replicate the reported study findings in their entirety. All PLOS journals require that the minimal data set be made fully available. For more information about our data policy, please see http://journals.plos.org/plosone/s/data-availability.

Upon re-submitting your revised manuscript, please upload your study’s minimal underlying data set as either Supporting Information files or to a stable, public repository and include the relevant URLs, DOIs, or accession numbers within your revised cover letter. For a list of acceptable repositories, please see http://journals.plos.org/plosone/s/data-availability#loc-recommended-repositories. Any potentially identifying patient information must be fully anonymized.

Important: If there are ethical or legal restrictions to sharing your data publicly, please explain these restrictions in detail. Please see our guidelines for more information on what we consider unacceptable restrictions to publicly sharing data: http://journals.plos.org/plosone/s/data-availability#loc-unacceptable-data-access-restrictions. Note that it is not acceptable for the authors to be the sole named individuals responsible for ensuring data access.

We will update your Data Availability statement to reflect the information you provide in your cover letter.

Our study’s minimal underlying data set is now publicly available on the Open Science Framework (OSF), along with the questionnaire (which was already available at first submission). We have included the link and the revised Data availability statement in our cover letter.

*Comment 5*

We note that you have indicated that data from this study are available upon request. PLOS only allows data to be available upon request if there are legal or ethical restrictions on sharing data publicly. For more information on unacceptable data access restrictions, please see http://journals.plos.org/plosone/s/data-availability#loc-unacceptable-data-access-restrictions.

In your revised cover letter, please address the following prompts:

a) If there are ethical or legal restrictions on sharing a de-identified data set, please explain them in detail (e.g., data contain potentially sensitive information, data are owned by a third-party organization, etc.) and who has imposed them (e.g., an ethics committee). Please also provide contact information for a data access committee, ethics committee, or other institutional body to which data requests may be sent.

b) If there are no restrictions, please upload the minimal anonymized data set necessary to replicate your study findings as either Supporting Information files or to a stable, public repository and provide us with the relevant URLs, DOIs, or accession numbers. For a list of acceptable repositories, please see http://journals.plos.org/plosone/s/data-availability#loc-recommended-repositories.

We will update your Data Availability statement on your behalf to reflect the information you provide.

There are no ethical or legal restrictions on sharing our de-identified data set. As explained in our response to the comment 4, our study’s minimal underlying data set is now publicly available on OSF. URL and references are in the cover letter.

*Comment 6*

Your ethics statement should only appear in the Methods section of your manuscript. If your ethics statement is written in any section besides the Methods, please delete it from any other section.

Please review your reference list to ensure that it is complete and correct. If you have cited papers that have been retracted, please include the rationale for doing so in the manuscript text, or remove these references and replace them with relevant current references. Any changes to the reference list should be mentioned in the rebuttal letter that accompanies your revised manuscript. If you need to cite a retracted article, indicate the article’s retracted status in the References list and also include a citation and full reference for the retraction notice.

Our ethics statement was originally in Participants subsection of the Methods section. We have moved it out of the Participants subsection and placed it at the beginning of the Methods section.

We also have thoroughly checked each reference.

2. Reviewer 1

*Comment 1*

Line 78 and 80: "or in Michigan" and "or in self reports". I believe the "or" in both instances are typos and should be replaced with "and"

The “or” in lines 78 and 80 have been replaced by “and”.

“Recent studies indicated that pandemic-related upheavals severely impacted the QoL and well-being of parents of autistic children in Northern Italy (10), in Turkey (11) and in Michigan (12); these results also emerged from a comparative study (13) and a scoping review (14). Surveys (15–17), parents and clinicians’ testimonies (18,19), and self-reports (20,21) further attested to the impact that lockdown had on autistic individuals.”

*Comment 2*

Line 102: the word "other" appears twice in this sentence. Please re-word the sentence to address this.

We have removed the first occurrence of “other” in line 102.

“In line with studies that addressed the impact of COVID-19 in other countries, our study targets the QoL of families confined with their autistic child (11) and potential changes in the behaviors of the autistic individuals (10,16).”

*Comment 3*

Line 142: Since data collection has been completed, please change sentence to past tense.

The tense of the sentence has been changed to the past tense.

“A parent questionnaire (implemented in LimeSurvey, 3.22.7 (37)), was made available online between the 20^th^ of April 2020, i.e. one month after the onset of the Belgian lockdown, and the 10^th^ of May 2020, date at which the full lockdown ended.”

*Comment 4*

Line 156: Please check for typo/grammar errors. e.g. "child" in this sentence should be replaced by "child's"

This typo has been corrected in the new version of the manuscript.

“The core of the study is based on the parental appreciations of changes in their child’s behavior and of their quality of life before and after lockdown.”

Please note that, following Reviewer 2’s comment, we have thoroughly revised the entire manuscript for typos and grammar errors.

*Comment 5*

Line 164: "in 15 subdomains" - should read "into 15 subdomains"

This change has been implemented.

“The questionnaire probed 3 fields (family characteristics, your autistic child, your QoL during lockdown) divided into 15 subdomains (home organization, job, resources, daily life at home, your child’s characteristics, your child’s sleep, your child’s diet, your child’s socio-communicative behaviors, your child’s progress, your emotional well-being, your material well-being, your physical well-being, your interpersonal relationships, your social connections, your personal development).”

*Comment 6*

Line 170 - 172: This sections, especially the part on 15 subdomains feels repetitive (ref Line 164). Consider re-wording.

The sentence has been rephrased in order to avoid repeating the information about the subdomains.

“The questionnaire was structured to facilitate completion and to make it easy for participants to take breaks.”

*Comment 7*

Line 173-174: Not sure if using a new paragraph was intentional, but feels unnecessary.

We have removed the jump to a new paragraph.

*Comment 8*

Line 221: "makes up the final sample.." . Please check the tense and ensure it is consistent throughout the paper and presented in past tense as the work has been completed.

We have changed this verb to past tense. As mentioned above, we have thoroughly revised the entire manuscript for typos and grammar errors, including tense mistakes.

“A total of 209 respondents made up the final sample.”

*Comment 9*

Table 1 is clearly presented and provides a good description of the sample, and variables explored. Nice work!

Thank you!

*Comment 10*

Line 326: Please use consistent terminology for this domain. I feel it is best to refer to it as labelled in a preceding section of the paper, "Home education strategies".

Thank you for this observation. The “home strategies” domain has been consistently labelled “home educational strategies” throughout the whole manuscript.

*Comment 11*

Line 369: I question if "paid a tribute" is the most appropriate wording to use in this context?

This is due to literal translation from French. The verb has been changed in the new version of the manuscript.

“Our results indicate that parental QoL, which is already fragile in families of autistic children (4–7), has worsen following lockdown restrictions, as 65% of the respondents reported a negative change in their QoL during the lockdown period, confirming previous findings that the lockdown had a negative impact on parents of autistic children (44).”

*Comment 12*

Line 469" replace "event" with "events"

This typo has been corrected.

“Information reported by the parents can obviously be influenced by their state of mind and recollection of events.”

*Comment 13*

Line 470: replace "rely" with "relied"

This typo has been corrected

“Additionally, key information such as diagnosis status could not be confirmed by a direct assessment and relied only on parental report.”

*Comment 14*

Line 472- 479: I question the relevance of this section as it is clear, a comparison with the counterfactual is not going to yield an answer to the research question. Consider if inclusion is therefore required.

This section has been from limitations subsection.

*Comment 15*

Line 482-484: Can the authors please consider including descriptors of the relevance of the findings to additional contexts, apart from when social contacts are restricted? Covid 19 disrupted routines, accessibility to support for caregiving etc. This concluding statement on the relevance of the findings should point to supporting families experiencing these scenarios as well.

We have included some clarifications about how the findings may generalize to other very common situations that families with autism have to deal with.

“Since this first lockdown, autistic people and their families had to get used to living with fluctuating and recurrent restrictions on social life. There is a need to pursue long-term follow-up of autistic people during and after the pandemic, and more generally, when their routines are disrupted (e.g., long summer breaks). In particular, there are outstanding questions on services, training programs and alternative ways (e.g., digital adaptations) to support populations with special needs when their social contacts are restricted or drastically modified when, for example, schools close during breaks or ends for young autistic adults.

*Comment 16*

The topic is interesting and relevant. The findings make an important contribution to the limited evidence base. The paper has been written exceptionally well, and was a pleasure to read.

Thank you again for your thoughtful comments and corrections.

3. Reviewer 2

*Comment 1*

Kindly describe how were the psychometric properties of the instrument ensured provided that some modifications were made to it.

Given the extraordinary and novel (at that time) circumstances of the pandemic, and the exploratory nature of this study which aimed at capturing immediate testimonies right in the midst of the first lockdown, we had to assemble an ad hoc questionnaire to fit our hypotheses. Parental quality of life was investigated with the Par-DD-Qol with only minor modifications intended to highlight the changes brought about by the lockdown. Other questions targeted classic socio-demographic variables, whose influence on the behavior of autistic people and families’ quality of life (i.e. co-morbidities, communication and language level, intelligence, challenging behaviors, access to support; e.g. Bitsika & Sharpley 2004; Cachia, Anderson, & Moore, 2016; Mugno, Ruta, D’Arrigo, & Mazzone, 2007; Rutherford et al., 2019). Finally, some additional questions targeted the specific context of the pandemic, for which no comparison was available at the time (acceptance of the restrictions, covid-induced anxiety…). It is worth noting that almost all other studies published on the same topic also used new questionnaires or surveys, whose validity and reliability have not been previously documented (Alonso-Esteban et al., 2021). This limitation is explicitly acknowledged as such at the end of the Discussion section. As explained in the limitations, the high internal coherence between the items targeting both quality of life and nonfunctional socio-communicative behaviors provides substantial validity to our instrument.

*Comment 2*

There are few but significant grammatical errors; thorough language-editing should be made.

We have thoroughly revised the entire manuscript for typos and grammar errors.
